# Temporomandibular disorder in construction workers associated with ANKK1 and DRD2 genes

**DOI:** 10.1590/0103-6440202204963

**Published:** 2022-08-26

**Authors:** Samantha Schaffer Pugsley Baratto, Michelle Nascimento Meger, Vânia Camargo, Gisele Maria Correr Nolasco, Natanael Henrique Ribeiro Mattos, Liliane Roskamp, José Stechman-Neto, Isabela Ribeiro Madalena, Erika Calvano Küchler, Flares Baratto-Filho

**Affiliations:** 1 School of Dentistry, UniDomBosco University Center, Curitiba, Paraná, Brazil.; 2 School of Dentistry, University of Tuiuti of Paraná, Curitiba, Paraná, Brazil.; 3 School of Health Sciences, Positivo University, Curitiba, Paraná, Brazil.; 4 School of Dentistry, University of the Joinville Region, Joinville, Santa Catarina, Brazil.; 5 School of Dentistry, Presidente Tancredo de Almeida Neves University Center, São João del Rei, Minas Gerais, Brazil.; 6 Department of Restorative Dentistry, School of Dentistry, Federal University of Juiz de Fora, Juiz de Fora, Minas Gerais, Brazil.; 7 Department of Orthodontics, University of Regensburg, Regensburg, Germany.

**Keywords:** Temporomandibular joint disorders, oral health, genetic polymorphism

## Abstract

The study aimed to explore the influence of genetic polymorphisms in ANKK1 and DRD2 on the signs and symptoms of temporomandibular disorder (TMD) in construction workers. This cross-sectional study included only male subjects. All construction workers were healthy and over 18 years age. Illiterate workers and functionally illiterate workers were excluded. The diagnosis of TMD was established according to the Research Diagnostic Criteria for TMD (RDC/TMD). Genomic DNA was used to evaluate the genetic polymorphisms ANKK1 (rs1800497) and DRD2 (rs6275; rs6276) using Real-Time PCR. Chi-square or Fisher exact tests were used to evaluate genotypes and allele distribution among the studied phenotypes. The established alpha of this study was 5%. The sample included a total of 115 patients. The age of the patients ranged from 19 to 70 years (mean age 38.2; standard deviation 11.7). Chronic pain (87.7%), disc displacement (38.2%), and joint inflammation (26.9%) were the most frequently observed signs and symptoms. The genetic polymorphism rs6276 in DRD2 was associated with chronic pain (p=0.033). In conclusion, our study suggests that genetic polymorphisms in DRD2 and ANKK1 may influence TMD signs and symptoms in a group of male construction workers.

## Resumo

O objetivo do estudo foi explorar a influência de polimorfismos genéticos em ANKK1 e DRD2 sobre os sinais e sintomas da disfunção temporomandibular (DTM) em trabalhadores da construção civil. Este estudo transversal incluiu apenas indivíduos do sexo masculino. Todos os trabalhadores da construção civil eram saudáveis ​​e maiores de 18 anos. Foram excluídos os trabalhadores analfabetos e analfabetos funcionais. O diagnóstico de DTM foi estabelecido de acordo com o *Research Diagnostic Criteria* para DTM (RDC/TMD). O DNA genômico foi usado para avaliar os polimorfismos genéticos ANKK1 (rs1800497) e DRD2 (rs6275; rs6276) usando PCR em tempo real. Testes qui-quadrado ou exato de Fisher foram utilizados para avaliar genótipos e distribuição de alelos entre os fenótipos estudados. O alfa estabelecido deste estudo foi de 5%. A amostra incluiu um total de 115 pacientes. A idade dos pacientes variou de 19 a 70 anos (média de idade 38,2; desvio padrão 11,7). Dor crônica (87,7%), deslocamento de disco (38,2%) e inflamação articular (26,9%) foram os sinais e sintomas mais observados. O polimorfismo genético rs6276 em DRD2 foi associado a dor crônica (p=0,033). Em conclusão, nosso estudo sugere que polimorfismos genéticos em DRD2 e ANKK1 podem influenciar sinais e sintomas de DTM em um grupo de trabalhadores da construção civil do sexo masculino.

## Introduction

The temporomandibular joints (TMJs) play crucial roles in mastication and jaw mobility, and verbal and emotional expression. Temporomandibular disorders (TMDs) include several disorders that can lead to orofacial pain symptoms ^1^. The etiology of TMD is multifactorial ^2^ and represents an interaction between physical, functional, and psychosocial factors ^3^. There is evidence that anxiety, stress, and other emotional disorders are directly related to TMD, especially in individuals who suffer from chronic pain ^2^.

The American Academy of Orofacial Pain introduced genetic factors as etiological factors associated with TMD in 2008. After this study, some studies in different populations and evaluating a variety of genes and pathways were performed. A systematic review from Visscher and Lobbezoo ^4^ reported that the literature mainly suggests genetic contributions from candidate genes that encode proteins involved in the processing of painful stimuli from the serotonergic and catecholaminergic systems. Another recent systematic review also supported that pain-related gene is involved in TMD ^5^. Two pain-related candidate genes are Dopamine Receptor D2 (DRD2) and Ankyrin Repeat and Kinase Domain Containing 1 (ANKK1). These both genes were also pointed as genetic predictors of human chronic pain ^6^. Variations in DRD2-mediated neurotransmission in central brain regions are associated with acute pain intensity in response to experimental pain stimuli in humans ^7^ ^,^ ^8^. This endogenous variation in DRD2-mediated signaling might be related to the individual genetic background, which affects DRD2 expression and function. Genetic polymorphisms in DRD2 have been associated with vulnerability to chronic pain conditions ^9^ ^,^ ^10^ ^,^ ^11^ ^,^ ^12^. DRD2 and ANKK1are both located adjacent to each other on chromosome 11q23.1 ^13^. The rs1800497 genetic polymorphism in ANKK1 can reduce dopamine receptor DRD2 expression by 40% ^14^, negatively influencing the dopaminergic pathway ^15^ causing a variety of phenotypes ^16^ ^,^ ^17^, including TMD-and pain-related phenotypes ^12^ ^,^ ^18^ ^,^ ^19^ ^,^ ^20^. Therefore, in the present study, we aimed to explore the influence of genetic polymorphisms in ANKK1 and DRD2 on the signs and symptoms of TMD and pain-related disabilities in a group of male construction workers.

## Material and methods

### Settings and Ethics

This cross-sectional study was executed in the Dental Clinic of the Social Service of the Civil Construction Industry Union of the Paraná State (SECONCI-PR), which is a state located in the south of Brazil. SECONCI-PR is a non-profit organization, linked to the Employer's Union of the Civil Construction Industry of Paraná that represents companies in the sector. The organization aims to promote health and safety at work for the construction workers in the region.

All the participants signed an informed consent form, and interviewers recorded their personal data after their approval. The study protocol was approved by the local Human Research Ethics Committees approved this study (number #2.802.708). The construction workers were consecutively included from 2018 to 2019. The Association study (STREGA) statement checklist was followed to develop and report the results of this study ^21^.

 Illiterate and functionally illiterate workers, those who were unable to understand and express themselves in written form, were not included. Women were excluded from this study. All construction workers were healthy adult males (older than 18 years old), that did not report to use analgesic medication or antibiotics in the past 6 months. The sample was composed only by workers employed in the physical construction.

### TMD evaluation

During the patient’s dental appointment, they were invited to participate in the project and answered the questionnaire. A senior dentist who had experience with TMD and was also trained and calibrated for diagnosis according to criteria of Axis I of the Research Diagnostic Criteria for TMD (RDC/TMD) performed the clinical examination. Axis I diagnose three groups of disorders: myofascial pain (with or without mouth opening limitation), disc displacements, and inflammatory conditions. Disc displacements by evaluated by side (left and/or right) with or without reduction. Inflammatory conditions were evaluated by side (left and/or right) and classified in arthralgia, osteoarthritis, and osteoarthrosis.

The RDC/TMD Axis II was filled by the patient to measure the pain intensity and the depressive symptoms levels. Chronic pain was graded from 0 to IV (0 - low incapacity; I - low intensity, II - high intensity, III - moderate limitation, and IV - severe limitation). Depression, Nonspecific Physical Symptoms Including Pain (NPSIP), and Nonspecific Physical Symptoms Excluding Pain (NPSEP) were classified as low, moderate, or severe.

### Allelic discrimination

Saliva samples from all included patients was also collected during the dental appointment and stored at -20ºC. The genomic DNA was extracted from the cells in the saliva using a method previously described was performed ^22^.

The selection of the studied genetic polymorphisms was based on their previous association with pain phenotypes. Therefore, one genetic polymorphism ANKK1 (rs1800497), and two genetic polymorphisms in DRD2 (rs6275; rs6276) were investigated. The characteristics of the selected genes and genetic polymorphisms are presented in table 1. The allelic discrimination was blinded and performed using real-time PCR (StepOnePlus™ Real-time PCR System) with the TaqMan™ assay (Applied Bio-systems, Foster City, CA, USA). A total volume of 3 μL (4 ng DNA/reaction, 1.5 μl Taqman PCR master mix, 0.075 SNP assay; Applied Biosystems, Foster City, CA) was used per reaction. The thermal cycling was set as follows: hold cycle of 95°C (10 min), and 40 amplification cycles of 92°C (15 s) and 60°C (1 min). Each plate had 2 negative controls. Internal consistency test was carried out by rerunning randomly 10% of all samples and presented 100% agreement.

**Table 1:** Studied genes and genetic polymorphisms

Gene	Position	Genetic polymorphism	MAF	Base change	Previous association
ANKK1	11q23.2	rs1800497	0.33	A/G	Associated with migraine susceptibility ^18^
					Associated with bruxism manifestation ^20^
DRD2		rs6275	0.47	A/G	Associated with acute pain severity after accident ^12^
					Associated with sleep tooth gridding ^20^
		rs6276	0.47	C/T	Associated with acute pain severity after accident ^12^
					Associated with sleep tooth gridding ^20^

### Statistical analysis

The Chi-square test was used to calculate the Hardy-Weinberg equilibrium. Chi-square test or Fisher exact test were used to evaluate genotypes (in co-dominant, dominant, and recessive analysis) and allele distribution among the studied phenotypes. Statistical analysis was performed using Epi info 7.1 using an alpha of 5% (p<0.05).

## Results

**Figure 1:**
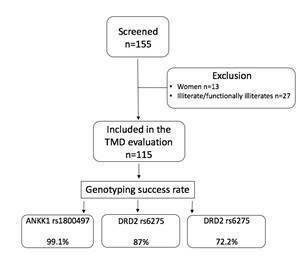
Flowchart of the study

The sample included a total of 115 male patients aged ranging from 19 to 70 years old (mean age was 38.2; standard deviation 11.7). The flow chart of the patient’s screening is present in Figure 1. Table 2 shows the prevalence of the Axis I and Axis II phenotypes. Chronic pain (87.7%), followed by disc displacement (38.2%) and joint inflammation (26.9%) were the most commonly observed signs and symptoms.

All the genetic polymorphisms assessed were within the Hardy-Weinberg equilibrium. Table 3 shows the genotype distribution of ANKK1 and DRD2 among the TMD signs and symptoms (phenotypes) according to the RDC/TMD Axis I and Axis II. The genetic polymorphism rs1800497 in ANKK1 was associated with myofascial pain in the dominant model, in which to carry two T alleles protect against myofascial pain (p=0.036; Odds ratio=0.13, Confidence Interval 95% 0.01 to 0.89). The genetic polymorphism rs6276 in DRD2 was associated with chronic pain (p=0.033), and the other genetic polymorphisms and phenotypes were not statistically significant (p>0.05). The allele distribution among the phenotypes were not statistically significant (p>0.05).

**Table 2:** Characteristics of the sample according to the signs and symptoms of TMD (RDC/TMD axis I and II).

Signs and symptoms	*n*	%
Myofascial pain	7	6.08
Disc displacement	44	38.2
Joint inflammation	31	26.9
Chronic Pain	100	87.7
*Depression*		
Normal	97	86.6
Mild	14	12.5
Severe	1	0.89
*Nonspecific physical symptoms including pain*		
Normal	103	91.1
Mild	10	8.8
Severe	0	0
*Nonspecific physical symptoms excluding pain*		
Normal	103	91.1
Mild	10	8.8
Severe	0	0

## Discussion

TMD is an important public health problem that affects between 5 to 12% of the population and is classified as the second most common musculoskeletal disease after chronic low back pain, resulting in pain and disability ^23^. The US study population from the National Health Interview Survey estimates the prevalence of TMD symptoms in women is twice that of men ^24^. Therefore, in the past few years, many studies have been focusing on the evaluation of the risk factors for TMD in women ^25^, while the studies evaluating males have been observed only in the studies that evaluate TMD in both genders. Despite the large body of research on sex differences in pain, there is a lack of knowledge about TMD related-pain in men. Additional to the sex differences, severity of TMD symptoms is also related to the age, in which older than 20 reports more frequently TMD ^26^. Additionally, construction workers spend many working hours at the workplace, which is an environment directly related to their health and well-being ^27^. In general, construction sites are known as a place with a high risk of injuries and poor health ^28^. Therefore, construction workers are predisposed to painful musculoskeletal problems and injuries because of their jobs. Thus, in our study population, only adult men were included in order to explore the candidate pain-related genes with the phenotypes of TMD.

A large cooperative agreement study called OPPERA (Orofacial Pain: Prospective Evaluation and Risk Assessment) evaluated the association of genes involved in pain processes with TMD. The majority of the genetic polymorphisms had stronger associations in women compared to men, but due to the smaller sample size of males the authors considered it inconclusive whether this trend is indicative of sex-specific genetic effects ^29^. In our population, we were able to observe a statistical difference for DRD2 rs6276 in patients with chronic pain and in ANKK1 rs1800497 for myofascial pain. 

Dopamine could cause joint pain processes, local sensitivity, and reflex activity developed by the muscles of mastication ^30^. A study that investigated subjects after motor vehicle collision also observed that rs6276 at DRD2 showed a significant association with pain scores ^12^. This polymorphism has also been associated with a manifestation of bruxism in children (sleep tooth grinding) ^20^. A recent systematic review and meta-analysis shows that both conditions may be associated ^31^.

The studied genetic polymorphism in ANKK1 was associated with myofascial pain. A previous study demonstrated that the rs1800497 polymorphism in ANKK1 is functional and can reduce the expression of the dopamine receptor DRD2 ^14^. The rs180049 has been linked to migraine susceptibility ^18^, as well as bruxism ^20^.

In our study, we used "Research Diagnostic Criteria/Temporomandibular Disorders" (RDC/TMD) instrument to diagnose TMD, which is a widely used tool in TMD research. This tool standardized the assessment and classification of patents. It is based on the biopsychosocial model of pain ^32^ and assesses physical disorder factors in its Axis I, while psychosocial factors are evaluated in the Axis II. Both Axis were used in our study.

**Table 3:** Genotype distribution according to the signs and symptoms of TMD (RDC/TMD axis I and II) and p-values of the comparisons for the genotype distribution in the co-dominant, dominant and recessive models and for the allele distribution.

	ANKK1 rs1800497						
	CC	CT	TT	*P-value*			
Signs and symptoms				Genotype			
*Myofascial pain*				Co-dominant	Dominant	Recessive	Allele
No	6(5.6)	42(39.3)	59(55.1)	0.052	0.051	0.287	0.146
Yes	0(0.0)	6(85.7)	1(14.3)				
*Disc displacement*							
No	4(5.7)	25(35.7)	41(58.6)	0.540	0.540	0.540	0.231
Yes	1(5.3)	10(52.6)	8(42.1)				
*Joint inflammation*							
No	3(3.6)	32(38.6)	48(57.8)	0.129	0.092	0.229	0.312
Yes	3(9.68)	16(51.6)	12(38.7)				
*Chronic Pain*							
No	1(7.1)	7(50.0)	6(42.9)	0.706	0.561	0.882	0.761
Yes	5(5.1)	40(40.4)	54(54.5)				
*Depression*							
Normal	5(5.2)	40(41.7)	51(53.1)	0.826	0.906	0.782	0.864
Mild	1 (7.1)	6 (42.9)	7 (50.0)				
Severe	0(0.0)	1(100.0)	0 (0.0)				
*Nonspecific physical symptoms including pain*							
Normal	5(4.9)	43(42.2)	54(52.9)	0.790	0.889	0.761	0.799
Mild	1(10.0)	4(40.0)	5(50.0)				
Severe	0(0.00)	0(0.0)	0(0.0)				
*Nonspecific physical symptoms excluding pain*							
Normal	5(4.9)	44(43.2)	53(51.9)	0.626	0.663	0.577	0.519
Mild	1(10.0)	3(30.0)	6(60.0)				
Severe	0(0.0)	0 (0.0)	0 (0.0)				

**Table 3:** continuation

	DRD2 rs6275						
	CC	CT	TT	*P-value*			
Signs and symptoms				Genotype			
*Myofascial pain*				Co-dominant	Dominant	Recessive	Allele
No	13(13.8)	48(51.1)	33(35.1)	0.581	0.721	0.999	0.662
Yes	0(0.0)	4(66.7)	2(33.3)				
*Disc displacement*							
No	11(18.0)	27(44.3)	23(37.7)	0.209	0.419	0.329	0.294
Yes	2(5.1)	25(64.1)	12(30.8)				
*Joint inflammation*							
No	11(14.7)	37(49.3)	27(36.0)	0.528	0.538	0.412	0.391
Yes	2(8.0)	15(60.0)	8(32.0)				
*Chronic Pain*							
No	0(0.0)	9(69.2)	4(30.8)	0.217	0.489	0.206	0.511
Yes	13(15.1)	43(50.0)	30(34.8)				
*Depression*							
Normal	12(14.1)	41(48.2)	32(37.7)	0.709	0.502	0.899	0.838
Mild	1(9.1)	7(63.6)	3(27.3)				
Severe	0(0.0)	1(100.0)	0(0.0)				
*Nonspecific physical symptoms including pain*							
Normal	13(14.8)	44(50.0)	31(35.2)	0.338	0.338	0.338	0.592
Mild	0(0.0)	6(60.0)	4(40.0)				
Severe	0(0.0)	0 (0.0)	0 (0.0)				
*Nonspecific physical symptoms excluding pain*							
Normal	12(13.5)	45(50.5)	32(36.0)	0.947	0.857	0.977	0.899
Mild	1(11.1)	5(55.6)	3(33.3)				
Severe	0(0.0)	0(0.0)	0(0.0)				

**Table 3:** continuation

	DRD2 rs6276						
	CC	CT	TT	*P-value*			
Signs and symptoms				Genotype			
*Myofascial pain*				Co-dominant	Dominant	Recessive	Allele
No	17(21.5)	37(46.8)	25(31.6)	0.479	0.321	0.576	0.566
Yes	0(0.0)	3(75.0)	1(25.0)				
*Disc displacement*							
No	13(24.1)	25(46.3)	16(29.6)	0.734	0.682	0.510	0.810
Yes	4(13.8)	15(51.7)	10(34.5)				
*Joint inflammation*							
No	12(18.7)	32(50.0)	20(31.3)	0.539	0.831	0.518	0.419
Yes	5(26.3)	8(42.1)	6(31.6)				
*Chronic Pain*							
No	0(0.0)	9(81.8)	2(18.2)	0.033*	0.719	0.489	0.710
Yes	17(23.6)	31(43.1)	24(33.3)				
*Depression*							
Normal	14(19.4)	34(47.2)	24(33.3)	0.570	0.472	0.762	0.693
Mild	2 (28.6)	3(42.9)	2(28.6)				
Severe	0(0.0)	1(100.0)	0(0.0)				
*Nonspecific physical symptoms including pain*							
Normal	16(21.9)	34(46.6)	23(31.5)	0.279	0.792	0.345	0.285
Mild	0(0.0)	5(62.5)	3(37.5)				
Severe	0(0.0)	0(0.0)	0(0.0)				
*Nonspecific physical symptoms excluding pain*							
Normal	15(20.1)	35(47.9)	23(31.5)	0.793	0.830	0.629	0.746
Mild	1(12.5)	4(50.0)	3(37.5)				
Severe	0(0.0)	0(0.0)	0(0.0)				

The idea is that other relevant patient characteristics that may influence the TMD ^33^. The RDC/TMD consists of a self-administered 31-question questionnaire and a 10-item clinical examination form, as well as clinical examination specifications and diagnostic criteria. The diagnosis can be multiple for a single patient. Axis I classifies individuals into three groups: muscle disorders (group I), disc displacements (group II), and other diseases such as osteoarthritis, arthralgia, and osteoarthritis (group III). While Axis II, divides them according to intensity and severity of chronic pain, degree of depression, and scale of non-specific physical symptoms. This multiple analysis performed due to the characteristic of the RDC/TMD could lead to a Type I error. However, in the present study, we decide to not perform any multiple analysis correction in order to perform an initial screen of the data. However, it is important to emphasize that future studies with a larger sample size in different populations should be performed to confirm our results. According to the Brazilian Institute of Geography and Statistics (IBGE, 2010) ^34^ the population of Curitiba has a specific ethnic composition: 78.8% are White/European descendants, 2.8% are Black/African descendants, 16.9% are bi-racial (Black and White), and the remaining 1.5% are Asian descendants or Native Americans, which differs from other populations, including from other areas of Brazil. An important note is that we did not stratify our analysis according to ethnicity, although some genetic polymorphisms could act as risk or protective factors according to the population, due to the high admixture of the Brazilian population, we decided to perform the analysis as a total group.

## Conclusion

Our study suggested that genetic polymorphisms in DRD2 and ANKK1 might influence TMD signs and symptoms in a group of male construction workers. More studies are necessary to confirm our findings.
